# Masson´s tumor in an uncommon location: A case report and literature review

**DOI:** 10.4317/jced.63653

**Published:** 2026-01-28

**Authors:** María Álvaro-Martinez, José Juan Pozo-Kreilinger, Luis Ortiz-Peces, Martin Andura-Correas, Guillermo Chacon-Ferrer, José Luis Cebrian-Carretero

**Affiliations:** 1 Department of Oral and Maxillofacial Surgery, Hospital Universitario La Paz. Paseo de la Castellana 261. 28046 Madrid, SPAIN; 2 Anatomia Patologica Hospital Universitario La Paz

## Abstract

Intravascular papillary endothelial hyperplasia (IPEH) is a rare, benign, non-neoplastic vascular lesion resulting from reactive endothelial proliferation associated with thrombus organization. Although uncommon in the oral cavity, IPEH may clinically mimic malignant neoplasms, frequently presenting as a nodular or ulcerated lesion and posing a diagnostic challenge. Histopathological examination remains the gold standard for diagnosis and plays a crucial role in the differential diagnosis, helping to avoid unnecessary aggressive treatment. Surgical excision with clear margins is generally considered the treatment of choice; however, evidence regarding alternative management strategies is scarce. We report the case of a 74-year-old woman who presented with an ulcerated lesion on the right lateral border of the mobile tongue, clinically highly suspicious for oral malignancy due to its morphology and patient-related risk factors. Histopathological analysis revealed intravascular papillary endothelial hyperplasia. Given the benign diagnosis, the patient's significant systemic comorbidities, severe cognitive impairment, and the high risk associated with surgical treatment and postoperative care, a conservative management strategy based on watchful waiting was deliberately chosen. No additional imaging studies were performed, as they were not expected to influence clinical decision-making. During follow-up, the lesion remained clinically stable without signs of progression. This case underscores the importance of considering IPEH in the differential diagnosis of tongue lesions and highlights that, in carefully selected patients, conservative management may represent a valid alternative to surgery, thereby preventing overtreatment of benign vascular lesions that clinically simulate malignancy.

## Introduction

Intravascular papillary endothelial hyperplasia (IPEH) is a benign pseudotumoral vascular lesion, also known as Masson's tumor or Masson's pseudoangiosarcoma, first described by Pierre Masson in 1923 as a "vegetant intravascular hemangioendothelioma" (1). The term IPEH was later introduced by Clearkin in 1976, who recognized the lesion as a reactive rather than a true neoplastic entity (2). IPEH accounts for approximately 2% of vascular tumors and most commonly occurs in the skin and subcutaneous tissues of the neck and upper extremities. Its presence in the oral cavity is rare, with only a limited number of cases reported. Within this region, the lower lip is the most frequently affected site, followed by the tongue and buccal mucosa (3). The lesion occurs more frequently in middle-aged women (4) and may clinically resemble hemangiomas, mucoceles, or angiosarcomas. Definitive diagnosis requires histopathological examination (4). Standard treatment consists of complete surgical excision with clear margins, yielding excellent outcomes and low recurrence rates. However, alternative therapeutic options such as sclerotherapy or beta-blockers have been proposed in selected cases (5). We present a case of IPEH of the tongue in a 74-year-old woman, discussing its pathogenesis, clinical presentation, and management, with particular emphasis on the decision to pursue conservative observation.

## Case Report

**Figure 1:**
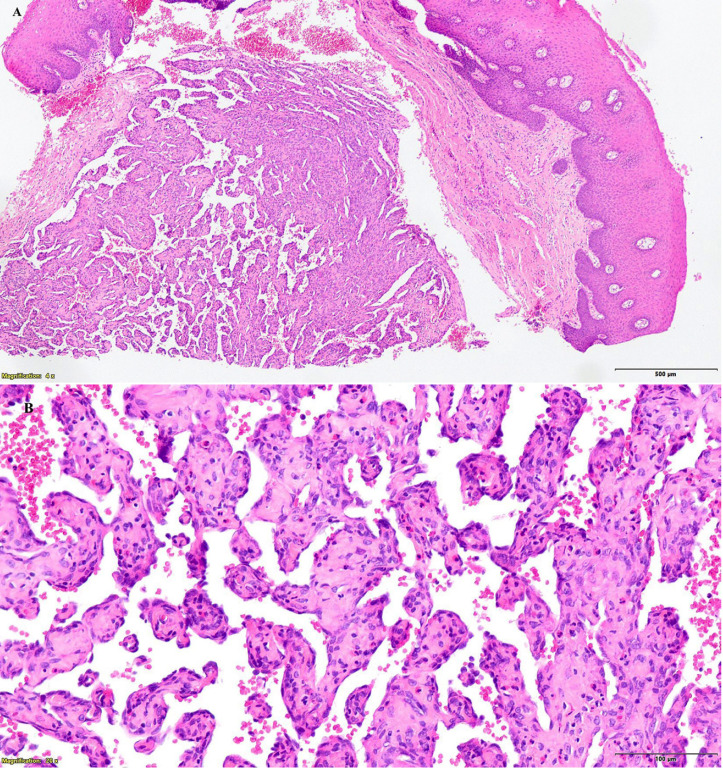
A) Light microscopy image, stained with H&amp;E. Vascular tumor within the lamina propria and submucosa, with intraluminal papillary projections. B) Light microscopy image, stained with H&amp;E. No mitotic figures or infiltrative behavior of the lesion are observed.

- Patient information A 74-year-old woman was referred to our Oral and Maxillofacial Surgery Department from her nursing home for evaluation of a painful ulcerated lesion on the right lateral border of the mobile tongue. The patient was functionally dependent and had a history of chronic alcohol abuse and long-term tobacco consumption. She suffered from alcohol-related dementia, which significantly limited communication, clinical examination, and the ability to establish the chronicity of the lesion. There was no reported history of local trauma. The patient had no previous history of malignancy, chronic infectious disease, or known vascular or coagulation disorders. - Clinical findings Intraoral examination revealed a red nodular lesion with a central ulceration located on the right lateral border of the anterior two-thirds of the tongue. The lesion measured approximately 1.5 cm in diameter, had irregular borders, was friable on palpation, and appeared adherent to the deeper planes. No additional mucosal lesions were identified elsewhere in the oral cavity. Cervical examination did not reveal any clinically palpable lymphadenopathy. - Diagnostic assessment Based on the patient's clinical background, risk factors, and the macroscopic appearance of the lesion, a malignant neoplasm of the oral cavity was initially suspected. An incisional biopsy was therefore performed. Histopathological examination revealed features consistent with intravascular papillary endothelial hyperplasia, characterized by papillary proliferations of endothelial cells confined within vascular spaces and associated with thrombotic material (Fig. 1).

1

No cytological atypia or infiltrative growth pattern suggestive of malignancy was observed. Given the benign nature of the lesion and the absence of clinical or histological signs of malignancy, no additional imaging studies or laboratory tests were performed, as they were not expected to modify the therapeutic approach. - Therapeutic decision and follow-up Surgical excision was considered; however, it was ultimately ruled out due to the patient's severe cognitive impairment, lack of cooperation, and inability to comply with postoperative care, which would have significantly increased the risk of complications such as infection or wound dehiscence. After multidisciplinary discussion and considering the benign diagnosis, the patient's comorbidities, and the overall clinical context, a conservative management strategy with close clinical observation (watchful waiting) was adopted. After 12 months of follow-up, the patient remains asymptomatic, and the lesion has shown no evidence of progression or malignant transformation.

## Discussion

Intravascular papillary endothelial hyperplasia is a rare vascular lesion that poses diagnostic challenges due to its clinical and histological similarities to malignant neoplasms. Most available evidence consists of isolated case reports and a limited number of systematic reviews, leaving several aspects of its pathogenesis incompletely understood. The most widely accepted theory describes IPEH as an unusual form of organizing thrombus. According to this hypothesis, an alteration or defect in thrombogenesis triggers an abnormal proliferative response of endothelial cells. The frequent association of IPEH with thrombotic material strongly supports this theory (5 , 6). Other authors have proposed a multifactorial etiology, including chronic minor trauma caused by local friction, as suggested by Gascón et al. (7). Furthermore, the higher incidence among women over 50 years of age has led to speculation regarding a potential hormonal influence, particularly the role of estrogens (8). IPEH lacks pathognomonic clinical features, making preoperative diagnosis difficult. It typically presents as a submucosal nodule or mass; in fact, approximately 90% of cases included in a Brazilian collaborative study and systematic review were described as nodular lesions (5). Most cases are asymptomatic, a finding consistently reported in the literature (4 - 6 , 8). This observation is consistent with the present case, as neither the patient nor her relatives were aware of the pre-existing nodular lesion. Despite its benign nature, IPEH may occasionally exhibit rapid growth or bleeding due to its vascular origin, further complicating clinical evaluation. Gascón et al. reported cases presenting with mass effect, paresthesia, or tooth displacement, leading to misdiagnoses such as angiosarcoma, squamous cell carcinoma, or melanoma (7). The most recent systematic review identified mucocele and hemangioma as the most common initial clinical hypotheses, each accounting for approximately 30% of cases (5). The differential diagnosis therefore includes virtually any oral mucosal lesion presenting as a mass or nodule (9). Table 1 summarizes the most frequently considered differential diagnoses. Given this diagnostic complexity, biopsy remains the gold standard.

Table 1

Histologically, IPEH is characterized by exuberant papillary proliferation within the lumen of an enlarged vessel, often associated with organizing thrombus, allowing differentiation from malignant entities, particularly angiosarcoma (5). Immunohistochemical studies are rarely required but may be useful in selected cases to confirm the vascular origin of the lesion. All cases identified in our literature review reported surgical excision as the treatment of choice. Alternative therapeutic options include sclerotherapy, laser therapy, or beta-adrenergic antagonists, following treatment protocols for vascular tumors (10). However, no previously published reports describe conservative management based on observation alone. In the present case, the patient's multiple comorbidities and social circumstances justified a watchful waiting approach. Although surgical excision is generally preferred, a surveillance strategy was adopted, and no lesion progression was observed after one year of follow-up. This outcome supports the concept of IPEH as a benign vascular lesion in which close observation may represent an appropriate management option in selected patients. IPEH is an important clinical simulator and should be considered when evaluating nodular lesions of the oral cavity. While surgery remains the primary treatment, patient-specific factors must guide decision-making. In the case presented, a conservative strategy proved to be a reasonable alternative and may help prevent overtreatment when histology confirms a benign vascular lesion with low-risk behavior.

## Figures and Tables

**Table 1:** Differential diagnosis of IPEH.

Hemangioma
Mucocele
Pyogenic granuloma
Fibroma
Vascular malformations
Salivary gland tumors
Squamous cell carcinoma
Angiosarcoma
Melanoma
Hemangioendothelioma

1

## Data Availability

The datasets used and/or analyzed during the current study are available from the corresponding author.
